# A Systematic Review on Pill and Medication Dispensers from a Human-Centered Perspective

**DOI:** 10.1007/s41666-024-00161-w

**Published:** 2024-02-22

**Authors:** Luigi Gargioni, Daniela Fogli, Pietro Baroni

**Affiliations:** Department of Information Engineering, University of Brescia, Via Branze 38, Brescia, 25123 Italy

**Keywords:** Pill dispensers, Medication dispensers, Healthcare technology, Patient-centered care

## Abstract

As medication adherence represents a critical challenge in healthcare, pill and medication dispensers have gained increasing attention as potential solutions to promote adherence and improve patient outcomes. Following the PRISMA (Preferred Reporting Items for Systematic reviews and Meta-Analyses) methodology, we carried out a systematic literature review on papers indexed in Scopus and PubMed, which present solutions for pill or medication dispensers. Given the importance of user acceptance for these solutions, the research questions of the survey are driven by a human-centered perspective. We first provide an overview of the different solutions, classifying them according to their stage of development. We then analyze each solution considering its hardware/software architecture. Finally, we review the characteristics of user interfaces designed for interacting with pill and medication dispensers and analyze the involvement of different types of users in dispenser management. On the basis of this analysis, we draw findings and indications for future research that are aimed to provide insights to healthcare professionals, researchers, and designers who are interested in developing and using pill and medication dispensers.

## Introduction

The global aging population is growing at an unprecedented rate [1], and as people age, they are more likely to develop chronic conditions such as diabetes, hypertension, and cardiovascular diseases. These conditions require ongoing management with medication therapy, which can be complex and challenging for patients. Patient non-adherence to medication therapy is a pervasive problem in healthcare, particularly among patients with chronic conditions living at home [2]. Non-adherence can occur for various reasons, such as patients forgetting to take their medication, misunderstanding dosing instructions, or intentionally choosing not to follow the prescribed regimen [3]. This can lead to poorer health outcomes, reduced treatment efficacy, and increased healthcare costs. Moreover, non-adherence can be especially concerning in home healthcare settings, as patients may not have the same level of support and supervision from healthcare professionals as they would in a hospital or clinic [4].

Scientific literature reports on a high variety of smart medication and pill dispensers aimed to provide patients with reminders for taking their medication at the correct time and to avoid errors about medicine type and dosage. Several solutions proposed in literature also envisage system architectures encompassing cloud services and mobile applications that allow caregivers and doctors to monitor patients’ medication adherence and manage the smart dispensers remotely. However, such solutions are still far from being widely adopted in real contexts, since several factors should be taken into account, including the direct connection with medicine prescriptions available in the national healthcare system, the management of complex therapies through automated devices without human intervention, the recharging of dispensers, their usability for elderly people, and, last but not least, the high costs.

The purpose of this paper is to carry out a systematic review with the objective of addressing the overarching research question:

*What kinds of automated dispenser solutions have been proposed in the scientific literature to help patients be adherent to prescribed care?*

In particular, given the fact that the adoption and actual utility of dispensers rely critically on acceptance and correct use by patients and other involved stakeholders, like caregivers and doctors, we adopt a human-centered perspective to address the above question. A limited number of literature reviews on this topic do not take this perspective, but usually deal with technological aspects of proposed solutions or are focused on specific patients’ diseases. For this reason, we aim to fill this gap in literature analysis by adopting a wider perspective that considers use context and interaction issues. Accordingly, we analyze the proposals in the literature with the main goal of identifying strengths and weaknesses that may affect acceptance and correct use by the people involved.

Following the PRISMA 2020 guidelines [5], we carried out a systematic review searching in journals, book chapters, and conferences through Scopus and PubMed, which led to the retrieval of 426 scientific papers, 56 of which were selected after the screening process and examined in depth for inclusion in this study.

The paper is organized as follows. Section 2 provides an overview of the related works; Section 3 details the methodology adopted for the systematic review; Section 4 presents the study’s results and addresses the four Research Questions into which the overarching research question has been decomposed; Section 5 delves into the study’s findings, current challenges, and implications for future research; and, finally, Section 6 provides some concluding remarks.

## Related Works

Few literature reviews have been published in the last years on the topic considered in this paper. Furthermore, they are usually focused on some specific aspects and rarely adopt a human-centered perspective.

The literature review reported in [6] does not follow a rigorous protocol for searching and selecting papers from the literature and does not present a clear research question; the authors would like to investigate the characteristics of wearable devices for measuring vital parameters and combine them with an automatic medicine dispenser; therefore, they report on solutions concerning different types of wearable sensors and medicine dispensers highlighting the most important technologies used in these solutions.

The paper [7] illustrates the systematic review carried out following a precise protocol described in a paper by the same authors [8]. The focus of this review is however pretty narrow: the authors are interested in collecting information and indications about the experience of informal carers of people with dementia using assistive technologies. Among the considered assistive technologies, there are smartphone applications, falls and motion detectors, smart gas meters, social assistive robots, and many others, including medication dispensers. Thus, on one hand, the analysis aims to acquire knowledge about carers’ experience and the impact of these technologies on their quality of life; on the other hand, medication dispensers are only listed among the assistive technologies mentioned in the various papers, without going into the details of their characteristics.

A scoping review carried out in 2019 is reported in [9]. The authors selected 85 articles out of 2638 for full-text review and scrutinized them focusing on the integration of smart multidose dispensing systems (SMODS) for medication adherence, where by integration they intended the usability, functionality, and acceptability of the system. Beyond a synthetic description of the proposed solutions, the review focuses on the data related to the different studies reported in the papers about medication adherence measurement and integration assessment. From the analysis, important gaps emerged related to the lack of a standard definition of medication adherence, an inadequate consideration of caregiver involvement in the studies, the limited feedback from healthcare professionals who play an important role in monitoring patients’ medication adherence, and the limited number of studies that considered the use of SMODS to manage complex therapies. The outcomes of this review are very significant from a process analysis viewpoint, but we would like to complement them with a socio-technical perspective, where both technical features of the proposed solutions in terms of hardware/software implementation and user-related issues, like management of the system on behalf of different stakeholders and interaction modalities, are considered.

A scoping review is also presented in [10], but it is focused on studies that involved people with a mental health or substance use disorder. An important inclusion criterion was that one technological intervention had been considered in the study to improve medication adherence; however, the authors define technology in broad terms, including mobile and web apps, computer vision-based systems, interactive voice response technology, smart pill bottles, ingestible sensors, and novel biomarkers. Smart pill containers are found in 34 studies out of 127 included papers, even though they are analyzed only in quantitative terms with reference to the evaluation of medication adherence.

The paper [11] presents a literature review on IT-based reminders, namely, different types of information technologies producing text, visual, or audio messages aimed at enhancing medication adherence. Particularly, the review analyzed 48 papers out of 544, including only those publications that quantify the effects of the considered reminder on medication adherence. A taxonomy is finally proposed that classifies the reminders according to 13 characteristics divided in four categories: reminder duration (long and short), reminder type (email, SMS, sensor/device, and pillbox), disease type (chronic, acute, and other), and medication adherence improvement (medium, small, zero/negative). Finally, the authors derive from the taxonomy a comprehensive framework leading to the development of medication reminders. This review can be considered complementary to ours, since it scrutinizes and classifies reminder solutions, which are typically included as an important feature in pill and medication dispensers.

The review presented in [12] has a goal similar to ours: providing an overview of current technologies to guide the development of a novel pill dispenser. This paper considers both commercial devices and devices proposed in scientific literature. Seventeen commercial devices are described in detail, selecting eight characteristics (dosage capacity, alerts to caregivers, device portability, peripheral communication, web or mobile app availability, security, multi-user capability, and power sources). As to the literature, papers were searched using Google Scholar and IEEEXplore with the same keywords that we in turn used to define our queries in Scopus and PubMed. However, the exclusion and inclusion criteria are not explained in the review, and only 16 papers (published before 2021) are synthetically described, among which 5 papers are published before 2013, 2 are not in English, 2 are only 2-page long, thus, all of them would have been excluded from our review; then, 2 papers are not indexed in Scopus or PubMed, and 3 papers were instead not retrieved with our queries in Scopus or PubMed; and only 2 papers are included also in our review, which reports extensive information on additional 54 papers, several of them (21) published since 2021.

## Methodology

Systematic reviews enable the creation of a comprehensive view of the scientific literature on a particular topic and an understanding of the open issues and challenges that may be related to it. In this review, we used the PRISMA 2020^1^ approach, guidelines, item checklist, and flow diagram [5].

While PRISMA is mainly intended for medical and clinical research, this study targets a wider audience, including computer science, human-computer interaction, and Internet of Things (IoT). As a consequence, for the sake of better comprehension by the reader, we have decided to modify the presentation and discussion order of the PRISMA items. Additionally, not all of the items were employed. The correspondence between the sections of this paper and the PRISMA items utilized in this study can be found in Appendix.

Another key difference in our application of the PRISMA guidelines compared to their use in clinical studies is that we did not differentiate between *Report* and *Study*, opting instead to solely employ the term *Paper*. According to PRISMA, *Reports* denote the actual scientific papers, while *Studies* refer to the research studies that led to specific results. As a result, PRISMA distinguishes between the research work as a whole and the publication of the results, as the results of a single study are frequently published in multiple papers. However, in our case, all 56 papers included in this review relate to distinct studies, obviating the need to differentiate between *Report* and *Study*.

### Research Questions

As outlined in Section 1, the central focus of this systematic review is to respond to an overarching research question: *What kinds of automated dispenser solutions have been proposed in the scientific literature to help patients be adherent to prescribed care?*

To guide our study, we defined four specific research questions:

- **[RQ1]**: What stage of development is the proposed solution at?
- **[RQ2]**: Which are the hardware and software technologies adopted in the proposed solution?
- **[RQ3]**: Which are the interaction modalities provided by the proposed solutions?
- **[RQ4]**: What kind of user involvement is envisaged in the proposed solution?

### Search Process

To include as much results as possible in our literature review, we used Scopus and PubMed. The former, which we adopted as primary source, is an online citations and abstract database.^2^ In the comparison with other digital libraries presented in [13], Scopus turned out to provide the maximum coverage. In particular, Scopus has an extended database, with tens of millions of records from tens of thousands of journals, conference proceedings, and book series. This breadth of coverage ensures that researchers have access to a diverse array of scholarly content from different fields and disciplines, which is essential when conducting a thorough and comprehensive review. About PubMed,^3^ it is a vast database comprising millions of biomedical literature citations from journals, books, and online sources. It covers a wide range of topics including medicine, nursing, dentistry, veterinary medicine, and health care systems. This is specific to the medical field, but to ensure relevant content is not omitted, research was also conducted on it.

We performed a query on Scopus on February 6th 2023 searching *“Pill Dispenser” OR “Medication Dispenser”* on the title, abstract and keywords. The only filter used was the English language. Following a suggestion provided by an anonymous reviewer, we checked our assumption about the coverage of Scopus by performing the same query on PubMed on October 23rd, 2023. The outcome confirmed our first assumption, since no further relevant papers emerged through this query, as it will be better explained in Section 4.

As for the time range, we chose the last 10 years, i.e., starting from 2013. This decision was based on the fact that the strong evolution of technologies quickly makes solutions in this field obsolete.

### Paper Selection

The title, authors, year, publication venue, and abstract of each retrieved paper were extracted and organized in a collaborative spreadsheet.

As to conference papers, we included full papers, short papers, papers presented at workshops, poster papers, late-breaking work papers, demo papers, and work-in-progress papers. We omitted workshop description papers, conference description papers, and doctoral consortium papers. We also excluded literature reviews so as not to report duplicate information already in individual papers. The discussion of literature review papers is however included in Section 2.

Table 1 displays a comprehensive account of the inclusion and exclusion criteria.

**Table 1:** The inclusion and exclusion criteria employed in this systematic review

Inclusion criteria	
IC1: Publication date between 2013 and Feb. 6th, 2023	
IC2: English language	
IC3: Conference papers types: full papers, short papers, poster papers, papers presented at workshops, late-breaking work papers, demo papers, work-in-progress papers	
IC4: Focus on a pill or medication automated dispenser and on the relevant technological aspects	
Exclusion criteria	
EC1: Conference papers types: conference description papers, workshop description papers, and doctoral consortium papers	
EC2: Literature reviews	
EC3: Low number of pages (less or equal to four pages)	

**Fig. 1:**
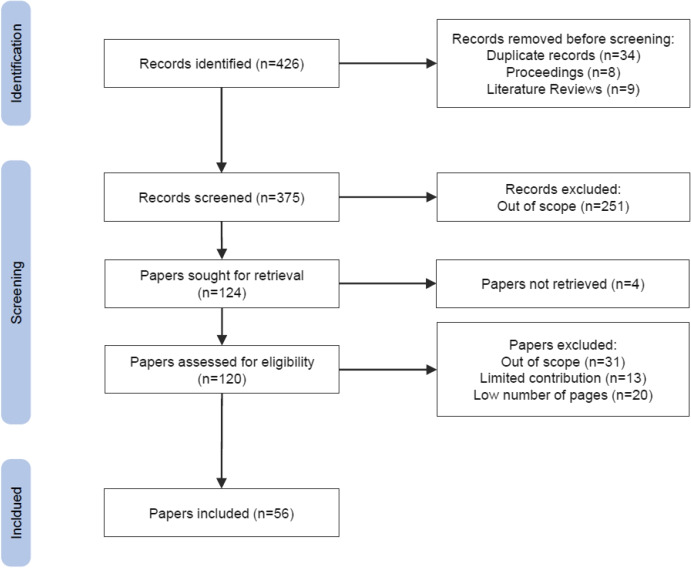
PRISMA flow diagram

Two reviewers conducted an independent screening of the papers to assess their eligibility for inclusion based on the predefined criteria. In case of discrepancies, the reviewers resolved them through discussion. Papers that did not meet the inclusion criteria were excluded, and the reason for their exclusion was documented in the shared spreadsheet.

A first screening step was performed by analyzing only the title and abstract of each document. Any discrepancies were discussed and resolved by mutual agreement.

**Table 2:** Papers included in the study classified according to the publication type

Type of publication	Number
Conference paper	41
Journal article	12
Book capter	3

Afterwards, the full texts of potentially relevant papers were obtained, and both reviewers screened them. The same approach as above was also used to resolve discrepancies in this phase. Inter-rater reliability was calculated for the full-text screening stage using *Cohen’s kappa* [14].

### Data Extraction

Additional columns were added to the spreadsheet to document all relevant data related to the research questions extracted from the included papers. Both reviewers thoroughly read and completed the spreadsheet independently. Afterwards, the two spreadsheets were compared, and any discrepancies were addressed through discussion until a consensus was reached by finally creating a shared spreadsheet.

## Results

The PRISMA flow diagram is reported in Fig. 1.

Out of the 426 papers initially identified (394 on Scopus and 32 on PubMed), 34 were duplicate papers, and 17 papers were excluded before the screening phase because they were descriptions of conference or workshop, proceedings, or literature reviews (see IC3, EC1, and EC2).

Of the 375 papers left, 251 papers were excluded after analyzing their abstract because they were considered out of scope (see IC4). Four papers could not be retrieved/downloaded.

The eligibility of the remaining 120 papers was assessed by reading the whole content, and 64 of them were excluded because they did not meet the inclusion criterion IC4 or met the exclusion criterion EC3. In this phase, *Cohen’s kappa* before reconciliation was 0.78. Table 2 shows the distribution of the selected 56 papers according to the type of publication, i.e., conference paper, journal article, or book chapter.

**Fig. 2:**
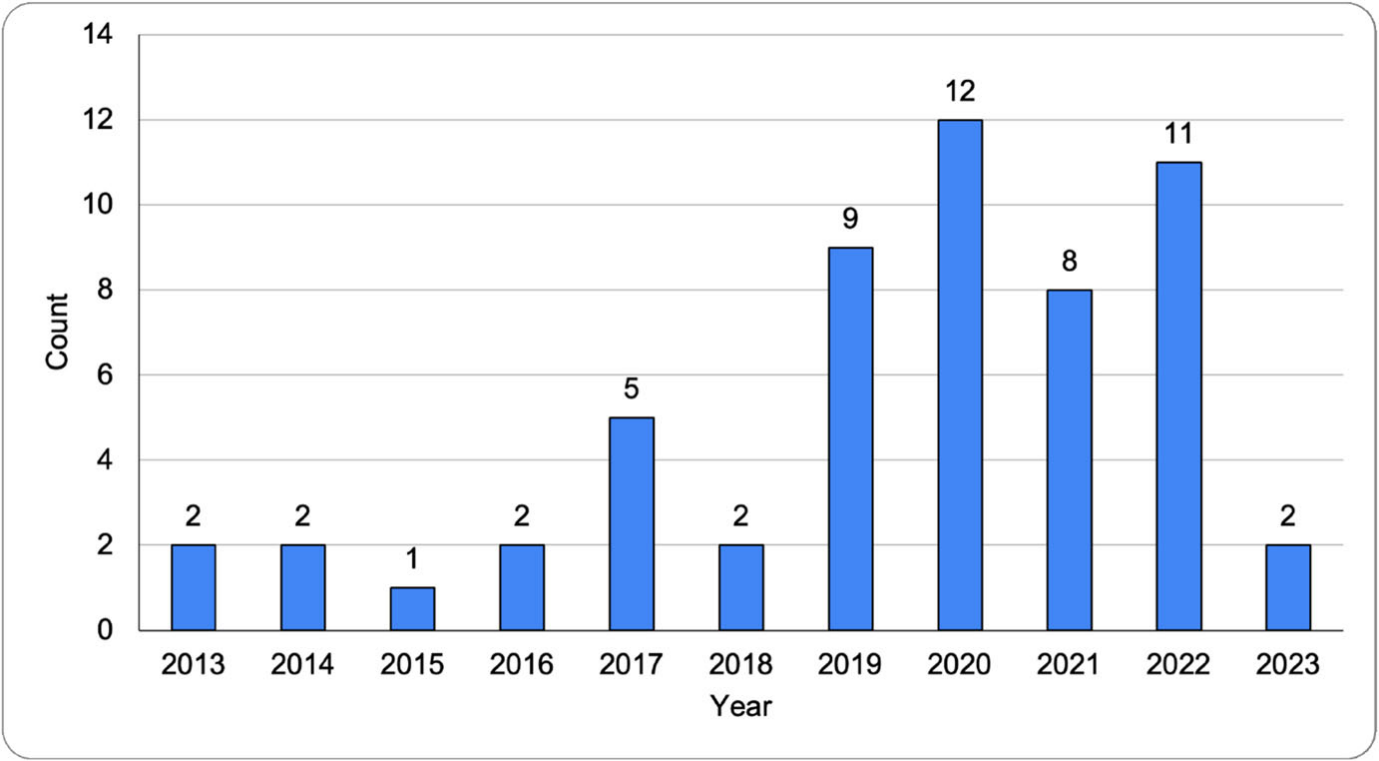
The number of papers published each year

Figure 2 shows the number of selected papers published each year from 2013 to the date of the query performed on Scopus (February 6th, 2023).^4^We may observe how the majority of papers (42 out of 56) have been published in the last 4 years.

For the sake of brevity, in the following sections, we will use just the word “dispenser” to denote “pill dispenser” or “medication dispenser.”

### RQ1: What Stage of Development is the Proposed Solution at?

As to the stage of development of dispensers discussed in literature (RQ1), we decided to classify the papers according to three main categories: (1) those presenting a design study of the dispenser or a very preliminary prototype; (2) papers illustrating the design, development, and (in some cases) evaluation of a working prototype; (3) papers discussing survey studies or comparative studies of commercial systems.

In the following, we provide a brief description of all the 56 selected papers, classifying them according to the above categories (see a summary in Table 3). To answer the other research questions, we will consider only the papers that provide the most significant information.

**Table 3:** Summary for Section 4.1

Stage of development	Total	Papers
Design studies and preliminary prototypes	35	[15–49]
Working prototypes of pill dispensers	13	[50–62]
Studies of commercial products	8	[63–70]

#### Design Studies and Preliminary Prototypes

Most of selected papers (35 out of 56) present a design study or a very preliminary prototype.

The work in [15] proposes the design of Medi-Deep, a comprehensive solution to address non-adherence problems. The architecture of Medi-Deep is outlined in the paper as a centralized system consisting of four components: the health record system (HRS) shared by hospitals, clinics, and pharmacies; the patient information system (PIS) that allows physicians to monitor if and what medicines are taken by a patient; the intelligent drug container (IDC) that is the hardware component at patient’s home; and the mobile tracking system (MTS), namely, a mobile application to provide patients with reminders and various types of information medications.

In [16], a comprehensive solution is delineated as well, which is specifically focused on patients with mild or moderate Alzheimer’s disease. Therefore, it includes as hardware components a simple dispenser and a set of alarm panels to be installed in house rooms to provide reminders through several alarm types. Different software components for the nurse/caregiver, the drugstore, the physician, and the hospital, all interacting with one another, are then hypothesized in the solution. Only preliminary prototypes of the medication dispenser and the application devoted to the nurse/caregiver are briefly presented in the paper.

A survey study and a design study are presented in [17]. As to the former study, state-of-the-art dispensers were first analyzed; then, 38 elderly people were requested to fill in an online questionnaire about their medication intake; finally, the usability of three commercial dispensers was qualitatively evaluated with 17 participants. For the latter study, 16 design variations of a modular dispenser concept were created and evaluated by experts of medical products. Three design concepts reached high consent, but their evaluation regarding the user interaction with the dispenser is left for future work, while functional and system requirements of an IoT-enabled dispenser and a preliminary hardware prototype are presented by the same authors in [18].

The solution proposed in [19] aims to overcome the issues of commercial dispensers existing at the time of paper writing (2019) concerning mobility, medication management, monitoring, updating medication schedule remotely, and authentication. It is an envisioned mobile dispenser designed for smartphones; it consists of software modules responsible for controlling and monitoring the dispenser, with data stored locally and on the cloud, and of a hardware module shaped as a mobile case with three cabinets locked with magnetic locks; the cabinets are opened only when attached to the smartphone, and there is a command to take or insert medicine.

A prototype of an IoT-based model of a multi-user dispenser using fingerprint authentication is presented in [20]; the paper reports the flowchart of the dispenser operation and a brief description of the developed hardware components.

In [21] and [22], proofs-of-concept medication dispensers are described by delineating their operation and the blocks composing the overall system; preliminary hardware prototypes with very limited functionalities are then presented. Similarly, [23] and [24] present the electronic components of basic and low-cost dispensers. The paper [25] describes the hardware architecture and operation of a medicine reminder and dispensing machine designed for people living in Indian local areas, who can only speech their local language; thus, the system includes an audio module recording reminders in the desired language.

The prototypes presented in [26] and [27] are preliminary as well, but, differently from the previous ones, they have been designed to send SMS messages to alert the patient when it is time to take medications; also, in these cases, a brief description of the components is provided, along with photographs showing their assembly. The low-cost characteristic of the proposed dispenser is highlighted in [28] and [29]: the former describes a crude implementation of a prototype, realized with local materials (wood and plastic), intended to be used in multi-person homes in developing countries; the latter illustrates how CAD software and 3D printing can be exploited to build a low-cost, strong, and light structure of the dispenser, even though its peculiar aspect is a mechanism to count the pills to be dispensed even in case of different pill dimensions. The designed dispensers are intended to be easier to use and cheaper than commercial ones, but no real experimentation is discussed. With a similar goal, the development of an automated dispenser for persons with neurodegenerative diseases living in low resource settings is presented in [30]; this simple and inexpensive device can automate counting and dispensing of medicines of different sizes with a pretty high accuracy, according to the system trials reported in the paper.

The dispenser described in [31], instead, is capable of automatically sorting pills within a container using algorithms for image recognition and optical character recognition (OCR); the work is however mostly focused on the evaluation of precision and accuracy of the algorithms, while it gives a brief account of the hardware and of the management application running on a personal computer. Image processing and OCR algorithms are also the main focus of [32], where the goal in this case is scanning prescriptions and then guiding the patient in filling the automatic dispenser; the device provides patients with reminders to take medications and doctors with information about patients’ medicine intake.

In [33], the block diagram and hardware components of a dispenser working as a reminder system for patients are described; its main novelty with respect to other proposed preliminary prototypes consists of a cooling system and storage compartments also for vaccines. Similarly, the hardware components of a simple dispenser are illustrated in [34, 35]; both papers also include a brief description of the Android application to be used to set the time slots for medicine schedule and keep track of pill taking. A mobile application is also part of the IoT-based medication management system that completes the dispenser proposed in [36]. In this case, the dispenser should serve different old residents in a center, and caregivers must use the app to manage medication treatments and receive notifications if patients do not take a medicine or the dispenser needs to be refilled. The proposal illustrated in [37] is focused on the cloud-based architecture of the dispenser, which allows patients, doctors, and pharmacists to monitor data about medicine intake and receive notifications.

Paper [38] reports on a smart dispenser combined with a pulse measuring system. The hardware prototype is delineated and shown with photographs, as well as the software component that consists of a web application for dispenser configuration and monitoring patients’ pill intakes. The dispenser presented in [39] includes a heartbeat and a temperature sensor; this device is simple, easy to use, and low cost, but its features are limited.

The dispenser proposed in [40] is meant to not only remind users to take medicine at the right time, but also to provide the correct pills and doses.A preliminary version of the system, developed using 3D printing and Arduino microprocessors, is briefly illustrated in the paper. Paper [41] investigates the role of IoT technology in healthcare applications, services, and products; then, the paper proposes a model of an IoT-enabled dispenser that is meant to be so simple and easy to handle that every elderly patient can use it easily; a cloud database accessible by the doctor would play an important role to store information about medication therapy and patients’ pill intake. The hardware components of a prototype dispenser are discussed in [42]; the original contribution of this work is the proposal of integrating this device in a smart wheelchair, but no testing or evaluation with users is presented in the paper. In [43], a syringe dispenser for diabetic patients is combined with a pill dispenser; the block diagram and a very preliminary realization of the system are then presented.

A modular dispenser is proposed in [44]; modularity is achieved by the number of boxes that contain the pills, which may vary according to the patient’s therapy and possible therapy changes. The solution is paired with a mobile application that allows therapy configuration and management and pill dispenser monitoring. A pilot study performed with five participants allowed to gather feedback on how to improve the mobile application.

In [45], the authors propose an automatic dispenser that encompasses a portable device and a fixed device that can communicate with each other through a USB cable; in particular, the portable device can retrieve the time and dose data of the medical treatment from the fixed device. The results of a 2-week experiment carried out with one female subject show how the subject improved her habit to follow the medical treatment. Notwithstanding these good results, the solution can be regarded as a proof of concept, since it requires several improvements in terms of performance, aesthetics, and integration with smartphones.

The preliminary prototype described in [46] is a moving dispenser that, beyond having features similar to those ones of other proposals in terms of hardware components and software application (i.e., a mobile app to set the time for medications), is able to deliver pills to the patient’s location, thanks to the implemented simultaneous localization and mapping (SLAM) algorithm. In [47], a different solution to medication adherence is proposed: the idea is designing and developing a mechanoid social robot able to detect the location of the patient through sensors (e.g., beacons) and dispense them the medicine into a container, by prompting a text message to the caregiver. To assess the feasibility of the idea, a lab-scaled prototype was developed using the LEGO Mindstorm toolkit. Performance tests were carried out on the prototype in different environments, and a user acceptance test was performed through an interview after explaining the technology. An autonomous robot to dispense medicines at the patient’s location is proposed also in [48]; it uses a mobile application to set the therapy, receive alert messages, and manage the interaction with the robot. The idea has been however only simulated at a software level. Similarly, [49] proposes a medicine dispenser robot for hospital wards and retirement homes, which can also carry out patient monitoring and voice assistance with prerecorded audio traces; a comprehensive solution is thus delineated, which includes an app for therapy management, a camera for patient surveillance and video recording, a temperature sensor, a speaker, and a microphone to acquire patient’s request.

#### Working Prototypes of Pill Dispensers

Thirteen out of 56 papers describe complete and working prototypes of dispensers that have been tested in terms of functionality and/or usability.

One of the first and almost complete research works is that presented in [50], where the authors adopted an iterative prototyping approach including phases of requirements analysis and concept design, prototype development, and user evaluation on the field. The personal medicine assistant is not properly a medicine dispenser, but a computer-based multi-modal reminder system that shows a medication plan and reminds the patients about the medicines to take with audio-visual information, and allows health professionals to plan the therapy and monitor medicine adherence. Two field trials, with four elderly people each and lasting 2 and 3 weeks respectively, provided satisfactory results about usefulness and user experience, as long as interesting feedback to improve the system.

The paper [51] illustrates the DIALCAT platform; it encompasses an IoT-enabled dispenser and a mobile application to obtain tailored information and communicate with doctors, caregivers, and pharmacists. The mobile application can also be used by caregivers and doctors to monitor the patient’s health and medication adherence and by pharmacists to receive orders to replenish the dispenser. At the time of paper writing (2019), a controlled clinical trial with 58 hospital patients in the control condition, 58 patients using the DIALCAT platform, and 58 patients who, beyond using DIALCAT, should have received periodic feedback on their metabolic control was planned.

In [52], the low-level details of the hardware implementation of a dispenser are discussed; then, a brief description of the software application where the user can insert the required time and the intake interval of each medication, and set related alerts, is provided. The paper also mentions a trial to assess the usability of the dispenser and its acceptability by patients, claiming that it helped geriatric patients remember to take their medications, thanks to its intuitive user interface; however, no specific information about this trial is provided in the paper.

The paper [53] describes a dispenser that, although very simple, can successfully remind patients to take their medicines via SMS and dispense pills of various sizes and shapes. The dispenser has been given to ten patients for a long-term test; after 10 weeks, a survey was conducted to collect information about the user experience, which resulted to be very satisfying, also because the system helped participants increase their medication adherence.

The authors of [54] explain how they extended the preliminary prototype presented in [28] to support patient-doctor remote communication, and report on a quality study based on key informant interviews performed with 17 health workers in four hospitals in Uganda; most of the participants considered the dispenser useful and easy to use, but they also suggested several improvements before actual deployment with patients could take place.

The work presented in [55] aims to develop a system that can automatically dispense pills of various sizes and shapes. The system, called PharmAid, consists of a portable wearable device providing reminders to users to take their medications and of a dispenser storing a month’s supply of pills. The inner pill container is able to move along the *x*, *y*, and *z* axes, and Bresenham’s algorithm is used to control the motors enabling the 3D movements; the paper is focused on its implementation in PharmAid and on the realization, assembly, and interaction of all the electronic components of the device. Simulation and stress tests contributed to study an improved version of the system that reduces dispensing time.

The paper [56] explains the design methodology and the hardware/software realization of a simple dispenser that rotates every 6 h making a pill drop in the container and triggering a buzzer to alert the elderly patient that it is time for the pill. Using Wi-Fi, the system also sends notifications to the caregiver and records the medication drop data for further analyses. The paper mentions user testing, without providing details about participants, and discusses possible improvements after tests.

The system proposed in [57] aims to combine IoT hardware and software technologies to create a system that encompasses a dispenser, a back-end software infrastructure, and a mobile application. The dispenser is modular and consists of five or more slots and can manage multiple users. A complete hardware and software system is proposed; a comparison with existing commercial dispensers shows its superiority in terms of costs and features. However, a usability and user experience evaluation has not been carried out. The same limitation affects the proposal presented in [58], where only functionality tests have been performed on the hardware device and mobile application developed. Interestingly enough, in this case, the mobile application is not only used to set up the therapy and monitor medicine intake, but it provides the patient with an easy way to operate the dispenser and obtain information from it, such as the number of pills left in the dispenser. A similar approach is followed in [59], where a dispenser for multiple users is presented; the paper mainly describes the mobile application used to set therapies, manage the dispenser, and send notifications to caregivers; also, in this case, tests concerned only system functionalities and not usability and user acceptability.

User tests with three participants have instead been performed on the smart medication planner (SMP) described in [60]. SMP is intended to help blind people manage their medication; thus, its architecture is studied to satisfy blind people’s needs, especially interaction based on voice communication. Even though SMP is still a prototype, its cost, portability, and usability resulted to be quite satisfactory for the users; however, some weaknesses were recognized such as the fact that voice recognition required a loud and clear voice and that the device packaging and size needed some improvements.

The solution proposed in [61] aims to cope with the problem of determining if the patient actually had ingested the medication taken from the dispenser; this is particularly important for patients with dementia. The authors propose a dispenser able to monitor the patient through sensors and to vocally guide them to complete the medication intake correctly. System evaluation (particularly activity recognition) was performed in a laboratory with 20 participants (faculty members and students aged 20 to 60) showing high efficacy of the proposed system; however, participants did not have dementia nor they suffered of health problems. The dispenser presented in [62] has been designed also considering patients who are paralyzed, deaf or blind, or have mental diseases, and is intended to be deployed in developing countries due to its low cost. The dispenser was tested by a female patient for 14 days at home; she had heart disease and a prescription to take medicines three times a day. Before the use of the dispenser, she forgot to take medicines at least 25% of the time, while, from data collected automatically by the system, it was observed that the forgotten rate decreased to 11.90%, with a significant difference between the first and the second week of dispenser usage (in the second week, the patient forgot only once to take medicines). Notwithstanding these good results, the experimentation with only one user is too limited to derive useful hints.

#### Studies of Commercial Products

Eight papers out of 56 present studies about commercial pill dispensers.

The paper [63] describes the evaluation in terms of acceptability and technical robustness of the Elucid Pill Connect system, a smart pill bottle dispenser. The use of the dispenser requires to interact with a dedicated mobile application. The study involved ten healthy participants that used the system with placebo capsules for 14 days. At the end, they completed the System Usability Scale (SUS) questionnaire, responded to additional satisfaction questions, and filled in the Medication Adherence Report Scale (MARS); in addition, they participated in a short interview to report problems that occurred during the trial and suggestions to improve the system. From the results, it emerged that, with this system, medication adherence was very high and participants judged the system acceptable from a usability viewpoint. Suggestions for system improvement include reminder personalization and additional features in the mobile app for training and accessing medication intake information. Finally, a couple of technical issues were found in the bottles to be considered by the manufacturer. The fact that the participants involved in the studied did not use prescribed medication, but volunteered to take placebo capsules, is the major limitation of this work.

The paper [64] describes an ethnographic study about the use of a robotic medication-dispensing system in the sparsely populated areas of Finnish Lapland. The system is an assistive technology reminding when medicines should be taken, which provides companion-like features. It was already on the market before the study took place. The study aimed at investigating how elderly people domesticate the system in their everyday lives and which meanings caregivers and health professionals assign to the robot during the domestication. The concept of *domestication* was used in the study, instead of adoption or use of technology, since it encompasses the meanings and values people assign to a given technology, how the technology is experienced, and which roles it plays in people’s everyday lives. The system dispenses tablet medicines on a regular basis; it can be remotely monitored and can be used for message exchanges between patients and the home-care service professionals. Eleven participants were involved in the study, including five elderly patients (73–89 years old), four nurses, and two other healthcare professionals. All the five patients lived alone and had impaired memory. Data from semistructured interviews and observations (with photographs) were collected along a 5-month period. One interesting outcome was that the patients considered the system as easy to use, but they could not use it without the nurses filling it with medicines regularly. Support for learning the system was also needed, but at the end, all patients were satisfied with the system, also thanks to the help of their social network (nurses and children). Some concerns about the system emerged related to its aesthetics and its inflexibility in dispensing medicines at specific times.

The paper [65] analyzes four commercial products—MedaCube, MedMinder, Hero, and Pria—that can dispense medicines automatically at the time predefined by the user (usually, a caregiver when the patient is an elderly person). Then, it presents a comparison based on design, pill refilling, services provided, sound and light alerts, security, and supervision features. Finally, an interdisciplinary team studied the user experiences in different settings (home environment, hospital environment, and nursing home) and arrived at defining the business model of a product, whose requirements, overall architecture, and basic device representation are described in [66].

In [67], the focus is on the evaluation of usability and task workload of dispensers, underlining how these aspects are critical to medication adherence of elderly adults. The paper reports on the quantitative findings (SUS (system usability scale) scores and NASA-TLX (NASA task load index)) of a study carried out in Canada, which examines 21 commercial products with the participation of 23 elderly adults (65 years old or older), five caregivers, and 11 health care professionals. Each participant tested five electronic medication adherence products, executing a series of tasks according to a mock polypharmacy medication regimen designed for the purpose of the study. The study demonstrates that some dispensers may be easier to use than others; however, the study has been carried out in an unfamiliar environment, without training, and with the request of using five different systems; this probably increased participants’ anxiety, frustration, and task load during the test. No design implication for the development of novel dispensers is provided in the paper. To overcome some of these limitations, in [68], the same authors present the results of a study about the use of the *Spenser* commercial dispenser at patients’ homes for a 6-month period. In this case, 58 patients and 11 caregivers were recruited, and usability, usefulness, and satisfaction were assessed through the SUS and the usefulness, satisfaction, and ease of use (USE) questionnaires, beyond medication adherence, impact on caregivers, and capability of pharmacists to identify problems. The results revealed a high mean medication adherence rate of (98%), and a very high SUS score (85.74); in addition, the dispenser allowed pharmacists to conduct medication reviews and identify drug therapy problems. Even though design implications are not reported here either, the study provides information to design future testing studies of similar products.

The paper [69] presents a randomized controlled study to compare the efficacy of Medido Connected, a commercial dispenser, versus regular care, in patients with Parkinson’s disease. Data were obtained in the Medido group and control group at baseline (36 vs 51 participants), after 3 months (24 vs 36 participants), and after 6 months (29 vs 45 participants) follow-up, by means of validated questionnaires assessing physical disability, quality of life, non-motor complications, and caregivers’ quality of life. A clinical improvement of physical disability in the Medido group was observed for the elderly and more severe patients. However, no significant differences were observed for the other patients. The authors hypothesize a negative influence on results of some limitations of the study, such as the many missing measurements at 3 months of follow-up (due to illness of the senior researcher), and the possible Hawthorne effect on the control group (improvements in therapy adherence may be due just to the inclusion in the study).

Finally, the paper [70] investigates the deployment of a medication management robot system (MMR) in home healthcare services of a municipality in Sweden, to understand the impact it could have not only on the elderly community, but also on health sector policymakers, elderly care managers, care personnel, and family members. The MMR involves a medicine dispenser that can administrate machine-packed dose sachets and a telecare system. The preparation of multidose sachets is carried out by the pharmacist who may access the system where the physician enters prescription information. Medicine sachets are delivered to a nurse; the nurse or their delegated home care person inserts the medicine sachets into the medicine dispenser and enters the medication information in the MMR software. The telecare system is used by the nurse and can monitor the MMR to alert caregivers if needed. A qualitative research approach (interviews, focus groups, workshops, and observations) is adopted in this study. Participants appreciated the system, which was perceived as easy to use, safe, and able to save time and costs for nurses dispensing medications themselves for patients. However, several interesting issues emerged from the study related to the organizational and inter-organizational complexity and the importance of information and communication quality. The paper describes these issues in detail and proposes further investigations on the topic.

### RQ2: Which are the Hardware and Software Technologies Adopted in the Proposed Solution?

In order to address RQ2, an examination was conducted to discern the hardware and software elements outlined within the various proposed solutions documented in the analyzed papers, with the aim of understanding their construction. To facilitate clarity and organization, this section has been subdivided into distinct categories, specifically *Electronics*, *Mechanics*, and *Software*, wherein the different components have been appropriately grouped.

#### Electronics

The main classes of electronic components we identified are microcontrollers, communication-related components, sensors, the real-time clock, and storage devices (see a summary in Table 4). Please note that this table and the following ones only include references to papers that contain information related to the table subject; papers where not relevant information is provided are omitted.

**Table 4:** Summary for Section 4.2.1

Electronic component	Total	Papers
Arduino	29	[16, 20–22, 25–27, 29–36, 38–40, 43–46, 48, 49, 56, 57, 59–61]
Raspberry Pi	10	[18, 20, 32, 34, 37, 41, 52, 57, 60, 66]
NodeMCU	2	[49, 62]
GSM	6	[26–28, 36, 53, 54]
Wi-Fi	14	[25, 34, 35, 39, 41, 44, 48, 49, 52, 53, 55, 56, 58, 60]
Bluetooth	9	[18, 22, 33, 40, 43, 46, 55, 59, 60]
NFC	2	[16, 50]
IR	11	[18, 20, 25, 27, 34, 36, 47–49, 51, 53]
Camera	6	[16, 31, 32, 40, 49, 66]
Kinect	1	[61]
Fingerprint	3	[20, 32, 33]
Real-time clock	4	[21, 23, 27, 53]
Storage	4	[21, 25, 44, 49]

##### Microcontrollers

Microcontrollers, such as Arduino, Raspberry Pi, and NodeMCU, are popular platforms used in the field of electronics and embedded systems. They provide a wide range of features and capabilities and offer different levels of computational power, flexibility, and connectivity options. The choice of the platform depends on the specific project requirements, complexity, and personal preferences of the developer. These platforms have vibrant communities and extensive online resources, making them accessible to both beginners and experienced developers alike.

A microcontroller can be used in a pill dispenser to control the dispensing mechanism and monitor the usage of the medication.

Arduino is used in most of the prototypes presented in the different papers. This is explicitly described in 29 papers, but the number could also be higher as in some papers no special attention was given to describing what hardware devices were used. Different Arduino models are used according to the computational power and compatibility requirements. The models used, where specified, are the *Mega*, *Uno*, and *Nano*. Using an Arduino in a dispenser prototype provides a flexible and cost-effective solution for designing a customized medication management system that can be easily adapted to meet the specific needs of different patients. In [20] and [25], the use of the Arduino within the proposed prototype is described in detail.

Raspberry Pi was used in ten prototypes. The hardware components in a dispenser, such as motors and sensors, can be connected to the Raspberry Pi through its GPIO pins. The Raspberry Pi can then be programmed to control the dispensing mechanism based on a schedule, triggered by sensors or user input. In addition to hardware control, the Raspberry Pi can provide a user-friendly interface for setting up medication schedules, monitoring dispensing events, and receiving alerts for missed doses. This interface can be connected to a display and input/output devices such as a keypad or touchscreen. Furthermore, the Raspberry Pi can store data on medication usage and potentially be integrated with other health monitoring systems such as wearables or health apps. This data can be analyzed to provide insights on medication adherence and potential health risks. The Raspberry Pi offers a versatile and cost-effective platform for building a customizable dispenser prototype with advanced data management capabilities. The use of Raspberry Pi is explained for example in [57] and [52].

Only two papers present NodeMCU as the main microcontroller. NodeMCU is a development board based on the ESP8266 microcontroller, designed for IoT. It offers an integrated Wi-Fi connection and a 32-bit processor and supports for the Lua programming language. In a dispenser, NodeMCU can be used to connect the device to a Wi-Fi network and send notifications to the patient or caregiver about the time of medication administration. In addition, it can be used to monitor the medication level inside the dispenser and to send alerts when a refill is needed. Wi-Fi connectivity also allows the dispenser’s data to be synchronized with a mobile application or web server, so that it can track a patient’s adherence to drug therapy and send reminders and alerts in the event of any problems or errors in medication administration. In [49], NodeCMU is used for a prototype of a dispenser equipped on board of a mobile robot, while in [62], it is used in a standard prototype as the main processor of the device.

##### Communication Components

Communication components play a vital role as they enable devices to transmit and receive data without a wired connection. Each technology has its own strengths and applications, and the choice of communication technology depends on factors such as range, power consumption, data rate, and the specific requirements of the project or application.

In six papers, a GSM module is included in the implementation. A GSM module is incorporated into a dispenser prototype to enable communication between the dispenser and a user or caregiver’s mobile device. Once the GSM module is connected, the pill dispenser can send text messages or make phone calls to a designated mobile number. This allows for remote monitoring and management of medication schedules, as well as alert notifications for missed doses or low medication levels. The use of a GSM module is central in [53] and [54].

The use of Wi-Fi is reported in the prototypes described in 14 papers. Wi-Fi can be used to connect the device to other applications or online services, allowing remote access and monitoring of the dispenser by caregivers or clinicians. An example of the use of Wi-Fi is presented in [56] and [49].

The use of Bluetooth is reported in nine papers. It is used to connect with a smartphone or tablet, which provides access to pill dosage information and the ability to change dispenser settings. In [43], this technology is used to make the main Arduino *Uno* microcontroller communicate with the Blynk IoT Platform for the exchange of the necessary information to manage the therapy. In the system presented in [46], it is used to connect the management app to the dispenser. In [55], Bluetooth creates a master–slave communication from the main device to the wearable one. The system proposed in [60] exploits Bluetooth connections to link the smart medicine planner with the smart medicine box. Furthermore, when the user requests to refill the medicine box, the program verifies the Bluetooth connection with it in order to dispense the medicine. In [40], the main Arduino controller receives the schedule of medication timing and type settings from a smartphone app through a Bluetooth wireless connection. The authors of [18] demonstrate the use of Bluetooth interfaces as a health gateway for acquiring vital signs; the dispenser is also equipped with alarm capabilities that communicate with the app. The exchanged data include the times when the box is opened and the reminder settings. The purpose of the study presented in [22] was to connect Bluetooth with an LCD display to facilitate the counting of pills in a tray, which could then be conveniently displayed on mobile phones or personal computers. Finally, in [59] and [33], the Bluetooth connection between the smartphone and the Arduino microcontroller allows the smartphone to transmit commands indicating which container and motor to activate, in order to dispense a specific pill from the container.

NFC technology is used in [50] and [16]. In the former, it is used to verify the medicine package, while in the latter, it is used to connect to the smartphone application of the nurse.

##### Sensors

In 11 papers, an infrared (IR) sensor is mentioned. The IR sensor is a hardware component that detects the presence of objects using infrared technology. In a dispenser, the IR sensor can be used to detect the presence of the user’s hand, so that the dispenser motor is only activated when the user is ready to receive pills or also to detect the presence of the pill in the compartment. For example, in [27, 51] and [36], this type of sensor is described.

In six included papers, a camera is used in the prototype implementation. In a dispenser, a camera can be used to verify that the patient has actually taken the pill, to recognize pills or prescriptions through image processing, or to create a direct communication with the patient. For instance, in [40], the proposed system incorporated a camera for capturing images of pills, which are subsequently transmitted to the app for recognition using convolutional neural networks. The solution presented in [31] uses the camera for OCR and medicine sorting, while in [32], it is used for scanning prescription through OCR. In [16], the camera is used to take shots of the moment in which the patient takes medication. In the solution presented in [66], the camera is used to make video calls with doctors, nurses, or caregivers, but also for facial recognition to access the system. The camera integrated in the system proposed in [49] is used for direct doctor assistance.

In [61], an approach for monitoring medical adherence via Kinect is presented. The Kinect is a motion sensor developed by Microsoft for the Xbox console. This technology uses a combination of cameras and depth sensors to track users’ movement and detect the position of objects in their surroundings. In this prototype, the Kinect is used to track the user’s movement while taking their medication.

In [20, 33] and [32], a fingerprint sensor is included in the described prototype. In a dispenser, it provides secure access control by using unique fingerprint patterns for user authentication. It enhances security and allows for monitoring medication usage and adherence.

##### Real-Time Clock

The real-time clock is a hardware component that keeps track of time precisely and continuously. In a dispenser, the real-time clock is used to keep track of time and date so that it knows when to dispense pills. The real-time clock can be programmed to activate the dispenser motor at specific times, ensuring that pills are dispensed at the right time. In addition, the real-time clock can be used to keep track of the last time the pills were dispensed, helping to prevent overdosing or underdosing. We assume that a real-time clock is included in all the prototypes presented, even when not explicitly mentioned. The description of this component is explicitly provided in four papers: [21, 27, 53] and [23].

##### Storage

In four papers, a secure digital (SD) card is used in the described implementation. In a pill dispenser, an SD card can be used to save pill dispensing data, allowing easy access and retrieval of information. The use of an SD card for the purposes just described can be found in particular in [44] and [21].

#### Mechanics

The mechanical components play a crucial role in enabling motion and physical interaction. The choice of mechanical components depends on the specific requirements of the application, including motion requirements, load capacity, precision, and environmental factors. In particular, several kinds of motors are used in the surveyed solutions.

A stepper motor is a type of electric motor that moves in a precise and predefined manner, step by step, when powered. These motors are often used in automation and robotics devices, where high precision is required. In a dispenser, the stepper motor is used to dispense the correct amount of pills. The motor is programmed to move the dispenser by a certain number of steps to dispense the desired dose. This is explicitly described in eight papers, for example, in [35] and [46].

In [23], a brush motor is used. It is a type of electric motor that uses brushes to supply power to the moving parts of the motor. These motors are typically used in small devices.

A servo motor is used in six prototypes. It uses a feedback system to maintain a specific position. These motors are often used in devices that require precise position control, such as drones or robots. The use of a servo motor is described for example in [43] and [48].

The motor driver is a hardware component that manages and controls the motor movements. It is a fundamental component, so most of papers do not describe it. In [42, 53] and [39], it is however explicitly mentioned.

#### Software

This section presents an overview of the software components adopted in the examined dispensers distinguishing software at the application level and at the infrastructure and system level (see a summary in Table 5).

**Table 5:** Summary for Section 4.2.3

Software component	Total	Papers
Android/web-based application	22	[15, 16, 18, 33–41, 44, 46, 48, 49, 51, 52, 57–59, 63]
Blynk IoT platform	2	[43, 56]
ThingSpeak	1	[56]
Cloud server	6	[18, 25, 37, 42, 51, 58]
MQTT server	2	[52, 58]
ROS	1	[46]

##### Application Level

At the application level, a mobile or web application is usually developed to manage and automate the process of medication dispensing. The application is designed to ensure medication adherence, organize medication schedules, and provide reminders to users. In addition, a cloud-based solution is often proposed to guarantee the storage and retrieval of information by different types of users (patients, caregivers, doctors, etc.). The application level for a dispenser focuses on creating a usable, reliable, and effective solution.

In 22 of the analyzed papers, an Android or web-based application is developed. An Android app is a software application designed to run on Android mobile devices. In a pill dispenser, an Android app can be used to monitor the status of the dispenser, display medication administration data, and send notifications to the patient or caregiver regarding the time of medication administration. In addition, the app can be used to synchronize the dispenser’s data with a cloud server or other applications. In several papers, it is not specified whether it is an Android app or whether it is a web-based application, but the functionalities provided by the two technologies are quite equivalent. Some papers describing the use of these platforms are [33, 34] and [39].

In [56] and [43], the Blynk App for smart homes is used. Blynk IoT platform is a platform that makes it easy to create apps for IoT devices and control them remotely. Blynk provides open-source code libraries and an intuitive user interface that allows app creators to drag and drop graphical elements to create customized interfaces. In the dispenser context, Blynk could be used to create a mobile app that allows users to monitor their medication dosage, set reminders, and keep track of their medication intake. In addition, in [56], the ThingSpeak service is used to record the medication drop data. ThingSpeak is an IoT service for collecting, analyzing, and visualizing data from sensors and devices connected to the Internet.

In six papers, the adoption of a cloud service is mentioned. A cloud server typically provides data storage and access to services through the Internet. In a dispenser, a cloud server can be used to store medication administration data, such as the time and amount of medication administered. In this way, the data can be easily accessed by the caregiver or the physician, who can check the patient’s adherence to the therapy and make any changes to the prescription. For example, the use of a cloud server is central to the projects described in [18] and [37].

##### System Level

By system level, we refer to components whose goal is the coordination and integration of other software and hardware components.

In [52] and [58], an MQTT (Message Queuing Telemetry Transport) service is used. MQTT is a lightweight and scalable messaging protocol used for communication between IoT devices. In a dispenser, an MQTT server can be used to send and receive data between the dispenser and other devices, such as an Android app or a cloud server. For example, the dispenser can send notification messages to the MQTT server regarding the time of drug administration, while the Android app can send commands to the dispenser to initiate the administration of a drug.

In [46], ROS (Robot Operating System) is used to control the mobile robot proposed as a dispenser. ROS is an open-source development framework used for creating robotic applications. It is used to manage sensors and actuators, such as proximity sensors to detect patient presence or actuators for drug administration.

### RQ3: Which are the Interaction Modalities Provided by the Proposed Solutions?

To answer RQ3, we analyzed the solutions proposed in the selected papers with the purpose of identifying the most common ways provided to end users (patients, caregivers, and health professionals) to interact with the device itself or access the data collected during its use.

A first classification of interaction modalities may consider (i) the solutions that offer a direct interaction with the hardware of the dispenser for operating on it, receiving reminders, checking its status, or recharging it and (ii) the solutions that provide a software application, running on a separate device, as an additional or alternative way to interact with the pill dispenser.

#### Direct Interaction with the Pill Dispenser

Several hardware components and related managing software have been included in the proposed dispensers. We list them in the following by considering the input components first, then the output components, and finally those components that support both input and output (see a summary in Table 6).

**Table 6:** Summary for Section 4.3.1

Direct interaction component	Total	Papers
Keypad and buttons	17	[22–24, 26, 28, 30, 35, 36, 38, 39, 54, 57, 58, 62, 64, 66, 70]
Microphone	3	[60, 66, 71]
Fingerprint	3	[20, 32, 33]
Facial recognition	1	[66]
LED	24	[15, 18, 21, 26, 27, 29–31, 33–36, 43–45, 51, 52, 55, 57, 58, 60–62, 66]
LCD	17	[22–30, 32, 34, 39, 41, 43, 46, 54, 56]
Speakers	13	[18, 21, 25, 28, 33, 42, 49, 57, 60–62, 64, 66]
Buzzer	19	[22–24, 26, 27, 30, 32–36, 41–45, 52, 55, 56]
Touchscreen	8	[29, 47, 50, 55, 64, 66, 68, 70]

##### Keypad and Buttons

In 17 included papers, the use of a dedicated keypad or physical buttons as a method of interaction with the dispenser is described. The buttons are used to activate dispenser functions, such as pill dispensing or cancellation of warnings. Some examples can be found in [22, 24, 62] and [58]. A keypad can be available to set medication timings, as in [22, 26] and [30], thus providing a simple interface, usually combined with an LCD, to interact with the dispenser.

##### Microphone

Only three solutions include a microphone. In [66, 71], we found that the microphone is used to make video calls with doctors, nurses, or caregivers, while only the dispenser described in [60], which is targeted to visually impaired people, exploits the microphone to help users take the right medicine at the right time. The interaction is however unnatural, since it consists of replaying system voice instructions obtained through pushing a button, and then the system recognizes users’ sentences using Google voice kit.

##### Fingerprint

Fingerprint authentication is a technology that allows the users to authenticate through the use of their fingerprints. In a dispenser, this technology could be used to ensure that only the authorized user can access the drugs contained within the dispenser. This technique is described in three papers: [20, 33] and [32].

##### Facial Recognition

Facial recognition is an alternative way for user’s authentication. For example, the user could register their face on the device, and the dispenser would use this registration to verify the user’s identity each time it is opened. In [66], the use of facial recognition to access the device’s system is mentioned.

##### LED

As to the output, the use of LEDs is the simplest and most intuitive way to signal something to the user. They are also often used because they are easy to install during the construction of a prototype. LEDs are used to indicate the status of the dispenser, such as whether the pills have been dispensed correctly or whether the dispenser needs to be charged. LEDs are mentioned in 24 papers, such as [18, 45] and [31].

**Table 7:** Summary for Section 4.3.2

Device interaction component	Total	Papers
Smartphone	28	[15, 16, 18–20, 26, 33–42, 44, 46, 48, 49, 51–53, 56–59, 63]
Personal computer	2	[31, 61]
Mobile robot	3	[46, 48, 49]
Wearable device	1	[55]

##### LCD

The LCD screen is used to display important information, such as the time the pills were dispensed, the number of pills remaining, the name of the medication, and the doses. In 17 included papers, the use of LCD is described, for example, in [29, 41] and [32].

##### Speakers

Speakers are another widely used way of providing dispenser output. They can be used to provide voice information, such as instructions for use, pill dispensing notifications, or low battery alerts. They also enable text-to-speech for visually impaired users, as in [60] and [33]. The solutions that include a speaker are presented in 13 selected papers, e.g., [28, 57] and [61].

##### Buzzer

The use of a buzzer is also widely used as a method to attract user’s attention, as it is very common in the current use of smartphones. The vibration can be used as a tactile but also as an auditory signal to alert the user of the need to take pills or for other notifications. In 19 papers, the use of buzzers is described, for example, in [27, 34] and [36].

##### Touchscreen

A dispenser endowed with a touchscreen provides the user with an easy and intuitive way both to obtain output information and to enter input in the system. It allows the user to select options, view information, and program the dispenser, according to doctor’s prescriptions. This type of interaction can be found in eight analyzed papers, for example, in [29, 70] and [64].

#### Interaction Through Separate Devices

As previously mentioned, several papers propose the use of a separate device, with a dedicated software application, to interact with the pill dispenser. The separate device can be a smartphone, a personal computer, a robot, or a wearable device (see a summary in Table 7).

##### Smartphone

The use of mobile applications or web applications running on smartphones is frequent in the selected papers (24 out of 56). They can represent the unique way to interact with the dispenser or can complement direct interaction with it. In the former case, the app covers all the offered functionalities, e.g., setting medication administration times, receiving notifications, and monitoring medication status, and it is used as well to activate the device, open/close its containers, and manage recharge. In the latter case, some of the functionalities are available through direct interaction with the dispenser (e.g., opening/closing containers). The implementation of mobile/web applications is described for example in [59, 63] and [51]. Notifications arrive as in-app notifications, informing the patient when it is time to take pills or if the dispenser needs maintenance, or alerting the caregiver/health professional if the patient has not taken the pills. In the solutions presented in [20, 26, 53] and [19], instead, notifications are sent to the user’s smartphone via SMS, without the use of any ad hoc mobile applications.

##### Personal Computer

In the solutions proposed in two papers, [31, 61], the interaction with the dispenser is mediated by the use of a personal computer where a specific software application runs and controls the device, possibly collecting all data about medication adherence and sending messages to the caregivers by email. The same software application can also be used to set medication doses and time schedule.

##### Mobile Robot

Three papers developed pill dispensers as mobile robots ([46, 48, 49]). For instance, [46] created a robot able to map the indoor environment and bring medicines to the patients; the goal was removing the need for human assistance especially during the COVID-19 pandemic. All the three solutions presented in the papers above combine the use of the mobile robot with a mobile application to set medication timings and control the robot.

##### Wearable Device

A wearable technology refers to a device that can be worn on the body, such as a bracelet or a watch. These devices are designed to collect and monitor data on the user’s physical activities, health, and daily habits. The wearable device can monitor medication data and send notifications to the user when it is time to take pills. In addition, the collected data can be sent to a caregiver or healthcare professional for remote monitoring. For example, paper [55] describes a wearable device that enables patients to receive reminders to take their medication even when they are away from the main unit. This is achieved by synchronizing the pill dispensing schedule from the main unit (master) to the portable device (slave). When it is time to take the medication, the portable device will emit an audible beep, vibrate, and flash an LED light.

### RQ4: What Kind of User Involvement Is Envisaged in the Proposed Solution?

To answer RQ4, we examined the solutions proposed in the selected papers considering whether they describe how the dispenser is recharged and how the medication therapy is managed. In particular, we focused on the role of users (patients, caregivers, doctors, nurses, and pharmacists) in these two important activities. Only a subset of selected papers is herewith considered, paying attention to those ones presenting the most interesting answers to RQ4.

#### Pill Recharge Management

Recharging a dispenser is a challenging task, since it must be performed following doctor’s prescriptions, avoiding contamination as much as possible, and managing the device component devoted to host the pills in an easy and safe way.

The different approaches to dispenser recharge are summarized in Table 8, even though several papers selected for this review do not discuss this problem at all.

**Table 8:** Summary for Section 4.4.1

Pill recharge methodology	Total	Papers
Manually by patient/caregiver	9	[23, 25, 27, 29, 36, 41, 45, 60, 62]
Manually, supported by software application	2	[38, 44]
Manually by healthcare professionals	1	[21]
Prepackaged medication bags	3	[64, 68, 70]

Most of the papers mention that the recharge unit must be filled with medications manually by the user, who might be the patient or their caregiver, e.g., [23, 25, 27, 29, 36, 41, 45, 60, 62]. For instance, in [27], it is explicitly stated that the dispenser must be preloaded by the patient or their caregiver once a day; the box where pills must be placed consists of several compartments, each one devoted to a pill to be taken at a definite time of the day. In one of the most dated proposals, described in [23], a caregiver must put pills in the system canisters, placing each set of drugs (up to 3) in a separate canister; the system also encompasses a pump to fill a glass with water from a water tank (that must be recharged manually as well), which will be drunk by the patient to take the pills; this latter feature is not foreseen in any other proposal. In the recent solution proposed in [62], medicines must be put in plastic pillboxes; it is possible to use up to three pillboxes per day, i.e., one for the morning, one for noon, and one for the night. Then, the pillboxes must be put in the device that has three columns corresponding to morning, noon, and night. When it is time to take the medicines, the user must press a device button, then the pillbox comes out; after the medicine is taken, the pillbox is re-inserted again in the dispenser.

A slightly more sophisticated solution, still requiring manual recharging of the pill dispenser, is that presented in [44]: here, a caregiver is required to use a mobile app to set up the therapy for the patient by associating each pill type and the number of pills to a different pill box forming the recharge unit of the pill dispenser; in fact, this solution offers the possibility to dynamically change the number of pill boxes according to the patient’s therapy; thus, once the pillboxes are physically set up and the therapy has been defined through the app, the last step is putting the pills in the boxes at regular times (e.g., every day, every week). A similar solution is implemented in [38], where a manual refill of three containers (corresponding to morning, afternoon, and night) can be carried out with the guide of a web application.

The boxes of the dispenser presented in [21] must be refilled by certified pharmacists following prescriptions and timings defined by doctors; this is considered an aspect of the prototype to be improved, by developing a proper software application to reduce the complexity of the refilling process.

A more automatic and controlled approach is that proposed for example in [68]: in this solution, medications are packaged in multidose pouches by pharmacies and delivered to patients weekly or every 2 weeks; these pouches are then loaded in the medication dispenser to be dispensed at prescribed times, according to the patient’s therapy. A similar solution is adopted in [70]: unit dose sachets of medicines for 1 to 4 weeks are machine-packed and delivered to nurses or delegated home care staff, who are in charge of bringing them to the patient’s home and inserting them into the dispenser, along with entering the patient’s medication information in the dedicated software application. As to the robotic medication-dispensing system discussed in [64], nurses are in charge of refilling it with medication bags every 2 weeks.

#### Therapy Management

In the dispensers presented in literature, different kinds of users are involved in therapy setting within the device, monitoring of therapy adherence, and data analysis of medication intakes. This led to develop different kinds of approaches through which the users set therapy or access data (see a summary in Table 9).

**Table 9:** Summary for Section 4.4.2

Therapy management methodology	Total	Papers
Web application	4	[38, 41, 45, 53]
Mobile application	7	[35, 37, 42, 57–59, 63]
Cross-platform mobile application	1	[57]
Direct interaction	7	[20, 21, 23, 24, 26, 29, 36]
Personal computer	1	[61]
Prescription scan	2	[15, 32]

A web application is adopted in the solutions proposed in [38, 41, 45, 53]. For example, a simple web application, hosted by the microcontroller of the smart device, has been developed in [53] to help the user configure the pill dispenser entering the prescription details and other device settings (time, phone number, Wi-Fi network, etc.). Within the solution proposed in [38], the medical staff uses a web application for setting the timing and number of medicines to be taken by the patients; a dashboard is also available to allow monitoring medicine intake and other physiological parameters. The web application developed in [41] is a cloud-based solution, and its database can be accessed both by patients and doctors for monitoring the intake of pills; the doctors are also in charge of inserting medication doses in the cloud database that is directly connected with the controller of the dispenser and allows its correct operation.

Several solutions propose the use of a mobile application for therapy management (e.g., [35, 37, 42, 57–59, 63]). An Android mobile application has been developed in [35], which allows one healthcare professional to manage different patients with related pill schedules and receive notifications about medication intake; this mobile application uses Firebase as an intermediate database that the dispenser can access; the interaction with the dispenser by the patient thus makes data changes occur in Firebase and consequent update of the app status. Similarly, paper [59] describes an Android mobile application that provides patients with features for controlling the dispenser and managing the patient schedule and data. After the user logs in the app, this one displays the list of pills to be taken in a given day; when setting the therapy, the user will add pills and timings, as well as related alarms, and choose in what container of the connected dispenser the pills must be placed. With the app, the user can also manage dispenser refill, since the app provides information about the remaining pills. To take their pills, the user positions the smartphone with the app close to the dispenser for connection via Bluetooth; then, the smartphone sends signals to the dispenser about which container should be activated. Caregivers have a passive role in this solution: their phone numbers can be entered into the app, and they will be notified by SMS when patients do not take pills. An Android-based application is proposed also in [58]: it communicates with the dispenser through Wi-Fi and can be used by the patient to set medicine schedule and related alarms and monitor medicines available in the dispenser; the caregiver receives notifications by email in case the patient has not taken their pills.

The authors of [57] developed a cross-platform mobile application that provides two different user profiles: one for the elderly patient and the other for the caregiver. The patient can use the mobile app to control the pill scheduling and receive notifications when pills must be taken; in some cases, the patient is capable and allowed to change pill scheduling and recharge the dispenser with the help of the app. Using the app with the proper user profile, caregivers can manage pill scheduling, temporarily stop the dispensing of one or more pills, and receive notifications in case of non-adherence or when the dispenser must be refilled.

More rarely in the selected papers, the user must interact directly with the dispenser, using its touchscreen, touchpad, or other special buttons, to set up the therapy. The latter solution is adopted in [20, 21, 23, 24, 26, 29, 36]. For example, the dispenser proposed in [29] is endowed with touchscreen and a graphical user interface (GUI) that allow users to set medication timings and view important information. A mechanism based on interrupts is implemented: guided by the GUI, the user can set up to five interrupts that control the system time and send notifications for pill intake. The dispenser presented in [26], instead, exploits an LCD display and a keypad to set the timings of medications. Similarly, the solution proposed in [24] has an interface consisting of an LCD and a keyboard that support the user in entering medication timings and selecting elements from a preprogrammed menu; the dispenser can be programmed for up to four time moments per day, for 7 days.

A dedicated personal computer, connected to the dispenser, is the solution foreseen in [61], where a software application implements most of the functions to monitor the patients and interact with them through prerecorded commands. In particular, a medical adherence controller takes the medication therapy from a database and data from the storage unit sensors, generates control signals for the hardware components of the dispensers, sends messages to the caregiver by email, and updates the database with information about medication adherence. In this way, the application also supports caregivers to manage medications and review patients’ activities.

There are finally specific solutions, such as that proposed in [32], in which the dispenser is able to scan the prescriptions and store in a MySQL database all information about medicines and their dispensing timings. A similar approach, based on scanning prescription data, is adopted in [15].

## Discussion

**Fig. 3:**
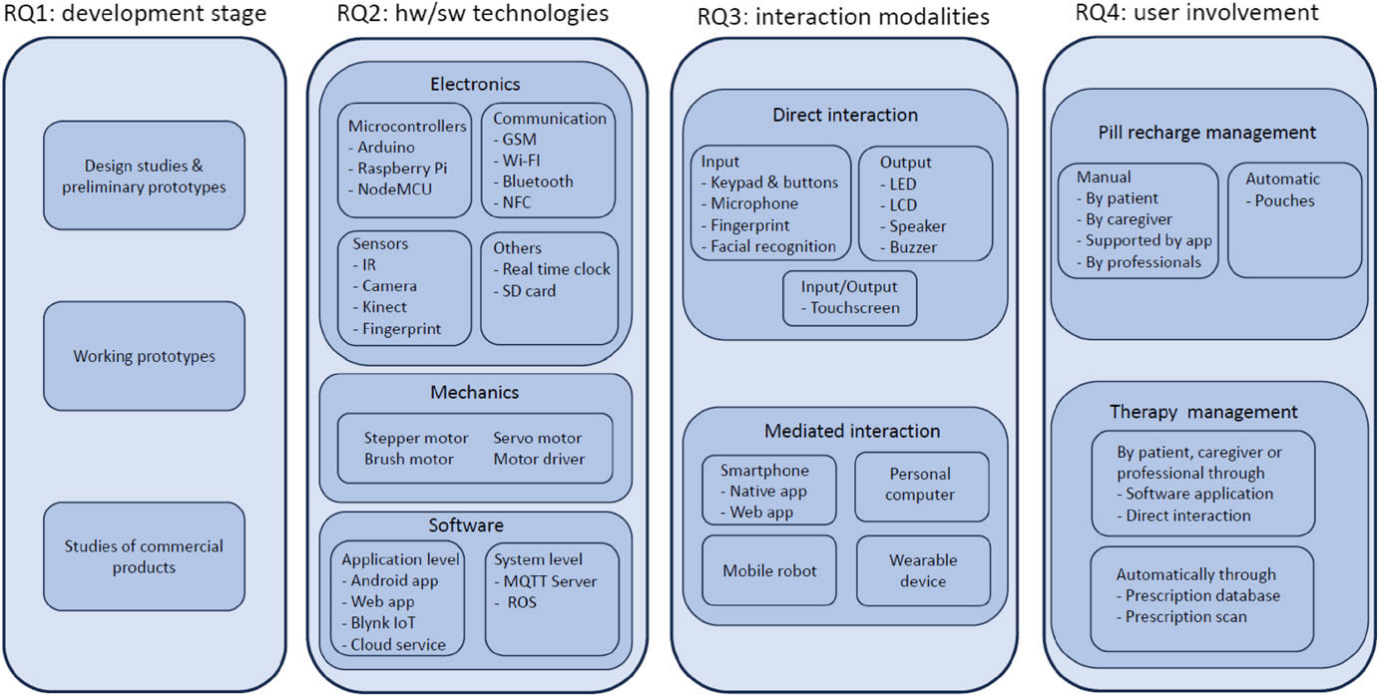
Classification of findings along RQ1-RQ4

In this section, we first summarize the findings emerged from the study (see Fig. 3), outlining the research gaps still present in the literature; we then discuss open issues and challenges to be considered while designing a novel solution in the field; we finally delineate implications for future research and describe the limitations of the research.

### Findings

Through our RQ1, we discussed the development stage of the proposals described in the selected papers, ranging from design studies and proofs-of-concept to working prototypes and comprehensive solutions that have been deployed and evaluated. It emerged that few papers present a user evaluation of working prototypes, while even fewer papers discuss field studies of complete solutions: this highlights how the research on automated dispensers is often focused on hardware and/or software technology, and somehow overlooks the impact on the various stakeholders (patients, caregivers, doctors, and the like), which is however crucial to achieve technology acceptance and relevant benefits at large. The few papers that describe some kind of system evaluation present user studies that involved a limited number of participants, usually only patients, while other stakeholders were more rarely involved. Among the eight papers that present studies about commercial devices (Section 4.1.3), only [64] adopts a comprehensive view on the problem by performing a long-term ethnographic study to investigate the impact of the technology on the everyday life of elderly people and their professional caregivers, while [70] collected qualitative feedback related to organizational complexity, and the other six papers limited their scope on testing the functionality and/or usability of specific devices. Furthermore, most of the papers do not describe a design methodology or architectural guidelines defining the roles and activities of the various stakeholders and the technical components that must be present in a comprehensive solution, thus limiting the chances for generalization and application in different contexts. Summing up, while there are many promising efforts, there are still significant gaps with respect to the development of mature solutions characterized by fully satisfactory user experience.

As to RQ2, findings were not particularly surprising. Papers describe the hardware and software components of their proposed prototypes, explaining their different features. Most of prototypes are based on flexible and low-cost microcontrollers like Arduino and Raspberry Pi and provide different levels of communication: from just sending text messages through a GSM module, to direct control of the dispenser through an application connected via Bluetooth, until remote access to online services and other IoT devices thanks to a Wi-Fi component. The latter is usually combined with a cloud-based software solution that contributes to create an articulated architecture, where the different stakeholders can access data according to their preferences and needs. This is crucial to create a comprehensive solution not focused only on pill dispensing. Therefore, the design and development of companion mobile or web applications become fundamental to increase the usability and acceptability of the dispenser for the different stakeholders. However, this type of architecture creates some dependencies, whose potential criticality is not discussed in the selected papers. In fact, dispenser operation will depend on the availability of external resources like Wi-Fi access and/or external software applications (at any level from smartphones to cloud services). Similarly, the integration of the dispenser in a richer IoT ecosystem, including sensors and wearable devices, may increase the number of features provided by the dispenser, but, at the same time, will add complexity to its management, including the programming of the behaviors of the IoT ecosystem, which may become a challenging task for patients, caregivers or other healthcare professionals. The endowment of dispensers with sensors for facial or finger recognition can be found in few proposed prototypes, even though this would significantly contribute to privacy and security. In a similar way, acquiring data about actual medication intake by elderly patients with cognitive impairments is a complex feature that is dealt with in a couple of selected papers, which provide however approaches aimed mainly at testing specific technologies devoted to this problem, rather than at integrating them within a complete solution to be deployed in real contexts.

The analysis of papers through the lens of RQ3 allowed us to identify different types of interaction modalities, from a simple operation on the medication dispenser to its configuration and control by means of a software application running on an external device. Surely, the latter solution offers more flexibility and scalability, even though it might increase the complexity of use: an elderly patient may find it more difficult to interact with the graphical interface of a mobile app to set the dispenser and read its notifications, than operating on the physical buttons, and being alerted by blinking LEDs and/or buzzer sounds. Natural language interaction via voice-based messages is proposed in very few papers, which consider dispenser accessibility for visually impaired users and in any case encompass text-to-speech technology or registered messages, while natural language understanding is not yet proposed for this type of system. None of the selected papers mention some tailoring possibility that concerns interaction modalities, to accommodate different patients’ attitudes and types of impairment (not only visual, but also hearing or cognitive). Papers that propose to adopt mobile robots to address the medication adherence problem are interesting, but at the moment, they are more suitable (and affordable) to healthcare structures than to use at home.

Through RQ4, we derived that there is a large spectrum of use contexts characterized by the involvement of different stakeholders in dispenser recharge and therapy management. As to dispenser preparation, it can be performed by the patient autonomously or by someone else close to them (i.e., an informal or formal caregiver), or preparation may be carried out in a separate site, like a pharmacy, by professional staff (e.g., pharmacists). Dispenser preparation is strictly related to the number of dispensers managed: in a home scenario, a single dispenser (or at most a small number of dispensers) is managed, while, in other scenarios (hospitals, retirement homes, and the like), a large number of dispensers needs to be managed together. Error prevention problems related to the latter situation are however not addressed in the selected papers. As to therapy management, the level of automation (and related cognitive effort) may vary a lot: since the dispenser is involved in a process that goes from therapy prescription, to planning medicine delivery and the relevant alerts, to the actual medicine intake, automated support can be present in all the process activities. For instance, prescription can be available in digital form and used to provide support for the correct dispenser filling and planning, or, alternatively, software applications (running on the dispenser or on a separate device) must be used for therapy definition; alerts can be used to remind of the moments when intake must occur, and some verification mechanism can be used to check that correct intake has occurred. While all these activities can benefit from automated support, in the selected papers, most of proposed solutions cover only some of them. Monitoring medication intake, if any, is supported in the majority of proposals through software applications that can receive notifications and provide information about patient’s behavior, while very few solutions exploit cameras and image processing algorithms to verify actual medication intake. Finally, none of the papers mentions which corrective mechanisms are put in place if the patient forgets to take one or more pills at a given moment; for example, a temporary revision of the therapy plan could be activated automatically by the dispenser or the problem could be submitted to the caregiver or doctor, possibly with some decision support provided by the system.

### Open Issues and Challenges

As evidenced previously, pill and medication dispensers represent a challenging subject, given their life-critical role, the variety of use contexts and stakeholders, and the possible involvement of fragile and/or impaired persons.

**Fig. 4:**
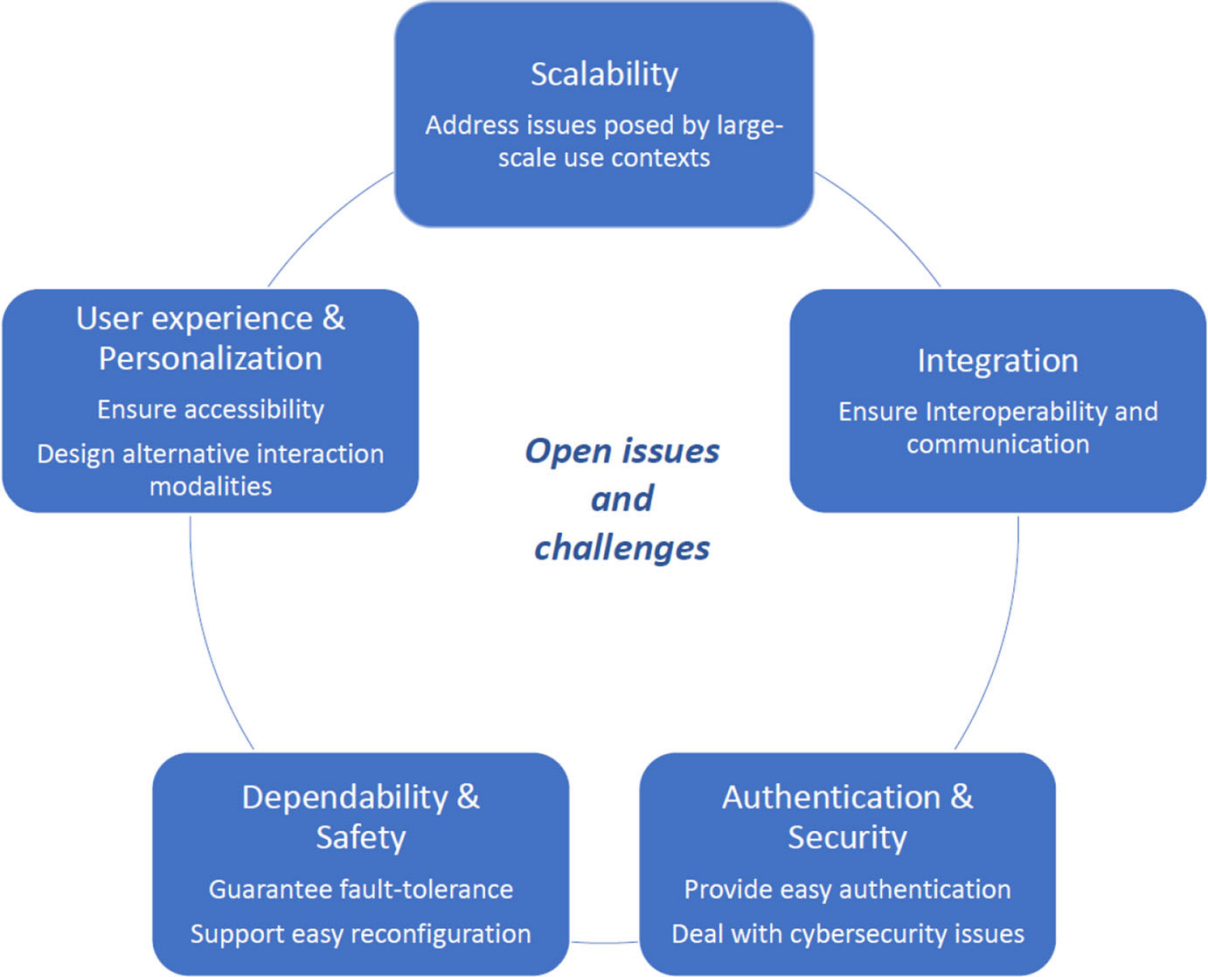
Summaries of open issues and challenges

As such, they are a lively and largely open investigation subject: while many studies and solutions have been presented in the literature, several important aspects appear to have received limited attention and require further developments, as listed below:

- *Scalability*: Small scale and in particular individual use scenarios are more typically considered as they represent the simplest use case and testbed for solutions which are still in their infancy. However, scalability issues need to be explicitly addressed in several respects:
  - Possible adoption of the technology by mid- and large-size institutions like hospitals and residential houses
  - In case some part of the process is not carried out at individual level, e.g., if many individual dispensers are prepared at pharmacies
  - In case some external resources (e.g., cloud services) are shared by a potentially large number of individual dispensers
  All the aspects in the design of a solution should undergo a scalability assessment. We remark in particular that if the phase of preparation and filling of the dispenser involves manual activities, they may represent a significant obstacle to scalability, as being slow and error-prone.
- *Integration*: The components and systems involved in the proposed prototypes, such as sensors, medication tracking software, and dispensing mechanism, are often developed independently and lack seamless integration. This lack of integration can lead to compatibility issues and limited functionality. It is essential for developers and manufacturers to focus on integrating these technologies effectively, ensuring smooth communication and interoperability among the different components. By achieving integration, they will be able to offer a better user experience, leading not only to improved medication management and patient care, but also to a more dynamic and open market for these solutions.
- *Authentication and security*: These aspects are not crucial during prototyping, but become critical in any actual deployment of the technology particularly in large-scale adoption scenarios. On the one hand, only authorized users should be allowed to interact with the dispenser. This is clearly crucial in contexts like hospitals, but is nevertheless important in domestic environments, e.g., in the presence of children. As obvious as this requirement is, it poses a challenge, since common authentication mechanisms may be difficult to use for elderly persons. On the other hand, especially if the dispenser is connected to the network, cybersecurity issues should be considered. Any attack potentially able to disrupt the dispenser operation may have life-critical consequences; appropriate prevention and mitigation measures should therefore be devised and put in place.
- *Dependability and safety*: A medication dispenser can be a life-critical device for some patients; hence, once the technology will be more mature, reliability and fault-tolerance issues will need to be the subject of suitable analysis, using consolidated engineering techniques [72] and possibly leading to consider some redundancy in the dispenser design. The ability of the dispenser to work in the absence of other components or services (e.g., in the absence of network connections) and the possibility of easy reconfiguration (e.g., of Wi-Fi settings) should also be considered. This is particularly relevant in the case where the dispenser must be moved from one place to another (e.g., when the patient needs to move for any reason including vacation, work, hospitalization). On the user’s side, safe usage considerations will need to be integrated in the interaction design, where the goal of providing a satisfactory user experience has to be balanced with the one of avoiding any unsafe operation.
- *User experience and personalization*: Physical and/or cognitive impairments of elderly patients are often underestimated, leading to the design of the interaction with pill dispensers based on graphical user interfaces (typically used in desktop, mobile, or web applications) which are unsuitable to patients’ special needs. With the growing diffusion of advanced paradigms, such as speech-based and gesture-based interaction, more appropriate ways to interact with dispensers could be studied. This opens up the challenge of designing different interaction modalities for the same device, so that the users could adopt that one they prefer and consider most suitable to their capabilities. This could also give rise to the need of implementing functionalities that allows caregivers or professional staff to easily tailor the user interface according to the patients’ characteristics and use context. Figure 4 presents a visual synthesis of open issues and challenges.

### Implications for Future Research

In addition to the specific challenges discussed in the previous section, we envisage two general indications for future work, which we regard as long-term research goals.

#### Flexible Design with Modular Architecture

The variety of possible use scenarios suggests that hardly a “one size fits all” approach is suitable for the design of pill and medication dispensers: the features that make a solution perfectly fit for a use case may be disadvantageous and prevent acceptance in another case. Patients with different degrees of autonomy, the variety of possible roles and competencies of caregivers, and the optional involvement of professionals like pharmacists and doctors are just some examples of the features that give rise to an extremely heterogeneous landscape in terms of user needs and hence of system requirements. Developing a babel of different products, each addressing a specific niche of use, does not seem a viable approach either.

We suggest therefore that, in order to enable the adoption of dispensers at large, a modular and context-adaptable approach needs to be investigated.

As a first step, one might undertake the identification of a set of basic functions common to any use scenario, e.g., providing scheduled reminders and dispensing pills and medications accordingly. These functions should be implemented by a core device, whose basic operation should be autonomous and robust, in order to ensure service continuity and also in case of failure of other components.

Then, a set of optional hardware or software components that can be added to the core device to enhance its capabilities should be designed in a modular fashion. To give a few examples, additional hardware components like a fingerprint reader might be devoted to user authentication while software components may extend the device functions (e.g., with automated therapy management) or support articulated scenarios with multiple roles (e.g., dispenser preparation by a pharmacist or therapy monitoring by a doctor).

To enable such a scenario, a suitable design methodology, oriented to openness and based on well-defined interfaces between components, should be adopted. In particular, we suggest that the functions of the core device should be associated with a standard set of application programming interfaces (APIs), whose definition represents a challenging task by itself. The broad adoption of such a standard would ensure, at least at a basic level, the interoperability between dispensers by different producers and device-independent software tools for dispenser configuration, control, monitoring, and so on.

While there is undoubtedly a significant gap between the current state of the art and the scenario envisaged above, we believe that stimulating research in this direction is crucial to overcome some critical obstacles to the adoption of dispenser technology beyond small-scale applications. Moreover, it would represent an enabling factor with respect to the more ambitious long-term research goal described next.

#### Towards Therapy Management Systems

The major goal of pill and medication dispensers is ensuring therapy adherence. However, a patient therapy may include aspects that cannot be managed by a dispenser. For instance, the use of an aerosol machine at home may be part of a therapy plan, and this cannot be reduced to the intake of something provided at the right moment by the dispenser.

Thus, the study of smart dispensers, as challenging as it is by itself, can be regarded as just a first step towards the more challenging goal of developing a patient-centered therapy management system of which the dispenser will be an essential, but not unique, component. In fact, this would require the realization of an ecosystem of therapy-supporting devices and of software tools for therapy planning, administering, and monitoring, along with the characterization of the various use contexts and of the roles of the involved people (mainly patients, caregivers, pharmacists, doctors).

Supporting technologies for such a scenario (in particular Internet of Things and cloud-based services) are available, while the main difficulty is to put together the pieces of the puzzle, whose complexity essentially lies in tailoring a set of technologies to the needs of the people they are intended to serve.

We believe that the development of human-centered projects, based on the use of dispensers, will represent a fruitful seed in this respect and can pave the way to the investigation of richer and more ambitious therapy management solutions.

### Limitations of the Research

This systematic literature review has the limitations indicated in the following. First, we limited our research to the period January 1st, 2013–February 6th, 2023, on Scopus (about 10 years). As already mentioned, the decision about the publication period was based on the observation that technologies for pill and medication dispensers rapidly evolve, making solutions obsolete very soon. In addition, we found that the most interesting proposals from a human-centered perspective have been published in the last 4 years. Then, we have had to limit paper search to the beginning of February 2023 to start paper analysis. At the date of submission, further papers addressing the topic of this study could have been published. However, as already mentioned, a further search on PubMed on October 23rd, 2023, did not find additional relevant work.

Second, our query string was based on the terms “Pill dispenser” and “Medication dispenser,” following the idea of the survey [12]. While we believe that these terms provide a very good coverage of the topic we aimed to address, we cannot exclude that in some other relevant papers, further and possibly more specific terms were used.

Third, our overarching research question only considered literature work; thus, we did examine only indirectly the existing commercial solutions available on the market, whose evaluation is reported in some of the selected papers (see Section 4.1.3). While other surveys on mobile health apps [73] have been successfully conducted resorting to app descriptions available online, in our case, analyzing from human-centered perspective commercial solutions not described in scientific publications would have required specific user studies, which were beyond the limit of the present paper. Surveys of mobile apps like the ones reported in [11, 73] are complementary to our work.

Fourth, the focus of our review was investigating the issues and challenges that could emerge from a human-centered perspective, by paying attention to the most interesting socio-technical aspects related to the development and deployment of medication dispenser technology. Other aspects like for instance the impact on therapy adherence of medication dispensers were out of the scope of our analysis and can be found in other literature reviews as indicated in Section 2. One of the possible outcomes of a systematic review is defining a taxonomy for a given research field (e.g., [11, 73–75]) which can be built from the findings in the reviewed papers following a suitable methodology, like the one proposed in [76]. Producing a taxonomy was not a goal of our work, which is focused on analyzing the human-centered perspective in the variety of proposed approaches to pill and medication dispensers. In particular, the requirement that the taxonomy considers characteristics that are common across all the articles [76] makes impractical the creation of a significant taxonomy in the context we considered, given the diversity of the surveyed papers. To address the difficulties arising from the requirement mentioned above, in [11], a comprehensive framework is proposed. In the same spirit, we provided a synthetic overview of our findings in Fig. 3.

## Conclusion

This paper presented a systematic review on pill and medication dispensers from a human-centered perspective. To this purpose, 426 papers indexed in Scopus and PubMed have been scrutinized, finding 56 papers relevant for our overarching research question. These papers have been analyzed considering the stage of development of the proposed solution, the developed software and hardware technology, the type of user interaction provided by the solution, and the type of involvement of different stakeholders in the use and management of the proposed solution.

**Table 10:** The description and position in this paper of the PRISMA items considered in the study are provided

Item	Description	Paper’s section
1	Identify the report as a systematic review	Title of the paper
2	Abstract	Abstract
4	Provide an explicit statement of the objective(s) or question(s) the review addresses	Introduction (Section 1)
5	Specify the inclusion and exclusion criteria for the review and how studies were grouped for the syntheses	Methodology (Section 3)
6	Specify all databases, registers, websites, organizations, reference lists and other sources searched or consulted to identify studies. Specify the date when each source was last searched or consulted	Methodology (Section 3)
7	Present the full search strategies for all databases, registers and websites, including any filters and limits used	Methodology (Section 3)
8	Specify the methods used to decide whether a study met the inclusion criteria of the review, including how many reviewers screened each record and each report retrieved, whether they worked independently, and if applicable, details of automation tools used in the process	Methodology (Section 3)
9	Specify the methods used to collect data from reports, including how many reviewers collected data from each report, whether they worked independently, any processes for obtaining or confirming data from study investigators, and if applicable, details of automation tools used in the process	Methodology (Section 3)
10a	List and define all outcomes for which data were sought. Specify whether all results that were compatible with each outcome domain in each study were sought (e.g., for all measures, time points, analyses), and if not, the methods used to decide which results to collect	Methodology (Section 3)

Item	Description	Paper’s section
16a	Describe the results of the search and selection process, from the number of records identified in the search to the number of studies included in the review, ideally using a flow diagram	Results (Section 4)
17	Cite each included study and present its characteristics	Results (Section 4)
23a	Provide a general interpretation of the results in the context of other evidence	Discussion (Section 5)
23d	Discuss implications of the results for practice, policy, and future research	Discussion (Section 5)

The findings show the existence of several gaps in the design, development, and deployment of comprehensive healthcare solutions that involve the use of pill and medication dispensers. The most important open issues and challenges have been derived from the analysis, highlighting how they concern solution scalability, system integration, authentication and security, dependability and safety, user experience, and personalization. As a final contribution of the review, two general indications for future work have been envisaged, suggesting the need for further research on design approaches to developing socio-technical solutions that may foster medication adherence in a broader sense, thus providing more comprehensive solutions for the improvement of patient health.

## Data Availability

The dataset of the papers analyzed for this manuscript is available from the corresponding author on request.

## Appendix. PRISMA items considered in the study

Table 10 lists all the PRISMA items used in this systematic review, along with their descriptions (as in [5]) and their corresponding location in this paper.

## Footnotes

1 PRISMA (Preferred Reporting Items for Systematic Reviews and Meta-Analyses): http://www.prisma-statement.org/

2 Scopus - Document search: https://www.scopus.com/

3 PubMed - National Library of Medicine: https://pubmed.ncbi.nlm.nih.gov/

4 As already mentioned, the query performed on PubMed did not provide additional papers.
